# Adiposity in Adults and Life Expectancy with and without Cardiovascular Disease. The Doetinchem Cohort Study

**DOI:** 10.5334/gh.1558

**Published:** 2026-05-26

**Authors:** Silvia Juliana Trujillo-Cáceres, Annelot P. Smit, Vicente Artola Arita, Marilyne Menassa, H. Susan J. Picavet, Klodian Dhana, Oscar H. Franco, W. M. Monique Verschuren

**Affiliations:** 1 Department of Global Public Health and Bioethics, Julius Center for Health Sciences and Primary Care, University Medical Center (UMC) Utrecht, Utrecht, the Netherlands; 2 Center for Prevention, Lifestyle and Health, National Institute for Public Health and the Environment (RIVM), Bilthoven, the Netherlands; 3 Julius Center for Health Sciences and Primary Care, University Medical Center Utrecht, Utrecht University, Utrecht, the Netherlands; 4 Department of Internal Medicine, Division of Epidemiology, Rush Institute of Healthy Aging, Rush University Medical Center, Chicago, Illinois, USA

**Keywords:** adiposity, life expectancy, life tables, cardiovascular diseases

## Abstract

**Background:**

While life expectancy (LE) in the Netherlands has increased over recent decades, it is not always accompanied by good health, particularly among individuals with adiposity. As adiposity is a major risk factor for cardiovascular disease (CVD), previous studies have explored its association with LE and CVD burden, but findings remain heterogeneous.

**Objective:**

To assess the association between adiposity and LE in adults, with and without CVD.

**Methods:**

We used data from the longitudinal Doetinchem Cohort Study (DCS), including 2,323 participants aged 50–70 years (49% women). Adiposity categories were determined (low, increased, high and very high) based on the combination of body mass index (BMI) and waist circumference (WC) thresholds for overweight and obesity based on the AACE/ACE definitions. CVD and mortality were assessed via linkage to hospital and death registries. A multistate life table approach estimated total and CVD-specific LE using transition rates between three health states—CVD-free to CVD, CVD-free to death, and CVD to death—incorporating prevalences and adjusted hazard ratios (HRs) for adiposity categories by sex and CVD status.

**Results:**

LE did not differ between adiposity categories at age 50 in both men and women. Men with very high adiposity had a 2.6-year lower LE free of CVD compared to men in the low adiposity category (difference: –2.6 years, 95% CI: –3.6, –1.5), and lived more years with CVD (difference: 2 years, 95% CI: 0.9, 2.9). Men in any adiposity category lived more years with CVD than women in a similar category. In women, there were no differences in CVD-specific LE between the adiposity categories.

**Conclusion:**

Men in the very high adiposity category lived more years with CVD compared to those in the low category. These findings highlight the need for targeted cardiovascular prevention in men with very high adiposity to delay CVD onset and promote a healthy lifespan.

## Graphical abstract

**Figure:**
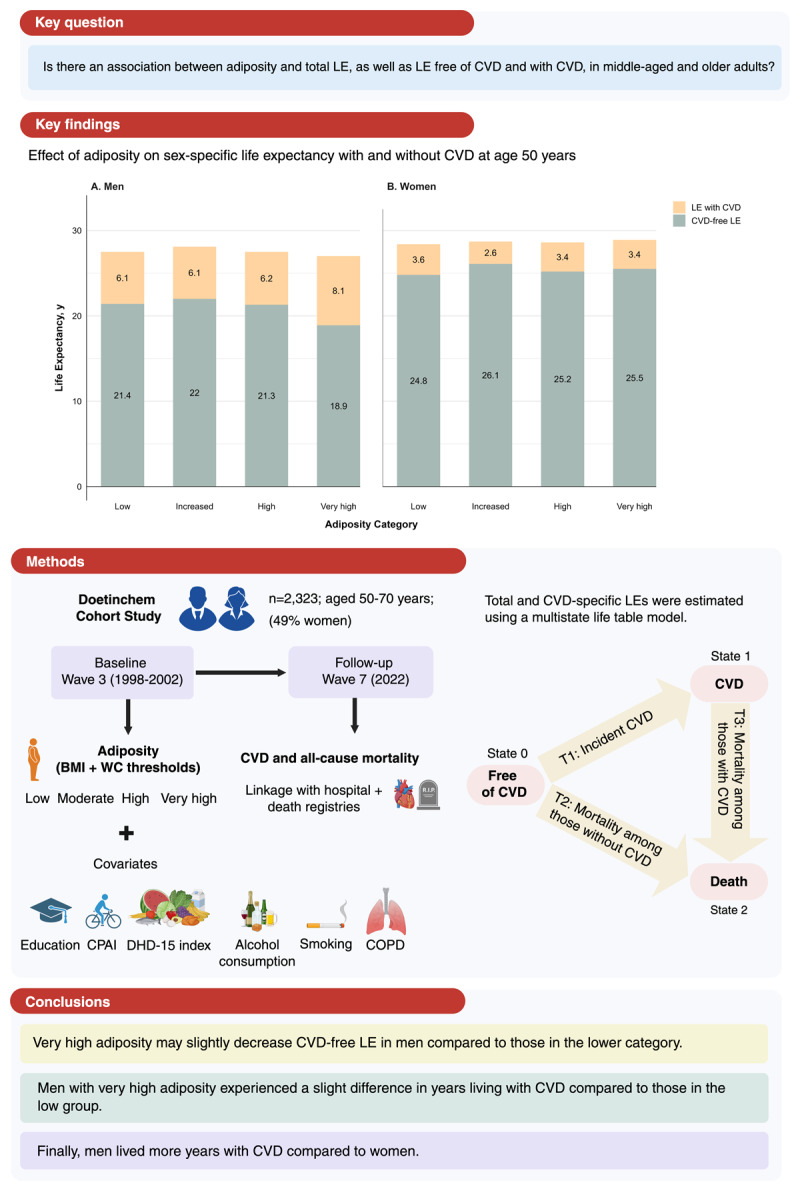
**Legend:** Estimated years lived free of cardiovascular disease (CVD) and with CVD at age 50, by sex and adiposity categories defined by combining BMI and WC thresholds (low, increased, high and very high). Final models were adjusted for age, household composition, education, physical activity, DHD-15 index, smoking (including cigarettes/day in current smokers), alcohol consumption and COPD. Created in https://BioRender.com. Abbreviations: BMI, body-mass index; COPD, chronic obstructive pulmonary disease; CPAI, Cambridge Physical Activity Index; CVD, cardiovascular disease; LE, life expectancy; DHD, Dutch healthy diet; WC, waist circumference.

## Introduction

Life expectancy (LE) at birth in the Netherlands has risen by approximately 10 years since 1950, to 80.1 years in men and 83.1 years in women in 2021 (1,2). However, these extra life years are not always spent in good health. Healthy LE can be influenced by lifestyle patterns that differ by sex (3) and by the growing burden of ageing-related morbidities and disabilities, including cardiovascular disease (CVD), diabetes, cancer and dementia (4,5,6).

Obesity is a major contributor to chronic disease and remains a public health challenge. Globally, an estimated 38% of adults are overweight or obese, with projections reaching 51% by 2035 (7,8). In the Netherlands, among adults ≥50–55 years, 38% are overweight, and 19% have obesity, with notable sex differences: overweight: 44% in men versus 32% in women; obesity: 18% in men versus 22% in women (9).

Previous studies have explored the effects of obesity on total LE (10,11,12) and LE with and without CVD, with heterogeneous findings (13,14,15,16,17,18,19). These heterogeneous findings might be explained by differences in calendar time, study populations, follow-up durations, methods for estimating LE and adiposity definitions. Body mass index (BMI), the most used metric, captures overall mass but not body composition or fat distribution, both of which are central to cardiometabolic risk, and change with ageing. Obesity prevalence has been observed to peak around age 60 and then decline (20,21); yet this pattern may not reflect a true reduction in cardiometabolic risk because BMI can miss age-related redistribution of fat.

Waist-related measures, such as waist circumference (WC), better capture visceral adiposity, which has strong metabolic and inflammatory effects. These measures may therefore reflect cardiometabolic risk more accurately than BMI alone (22,23,24,25). Furthermore, they are simple, reproducible and have shown associations with CVD outcomes. Large cohort studies have linked higher WC or waist-to-hip ratio to CVD and mortality; however, relatively few have quantified these associations in years of life lost (26,27). To our knowledge, no prior study has used a multistate life table model to estimate LE by joint BMI-WC categories that help to more accurately capture adiposity and transitions between cardiovascular health states.

Therefore, we aimed to assess the association of adiposity, defined by BMI in combination with WC, with total LE, and with LE free of and with CVD in middle-aged and older adults from a population-based cohort with more than 20 years of follow-up.

## Methods

### Study population

We used data from the Doetinchem Cohort Study (DCS), an ongoing, population-based longitudinal study conducted in the town of Doetinchem, Netherlands. The study was initiated between 1987 and 1991 to investigate the long-term impact of lifestyle and biological risk factors on health across the life course. At baseline, 12,405 individuals aged 20–59 years completed questionnaires and underwent physical examinations. A random subsample of 7,768 participants was invited to the second wave (W2, 1993–1997), with a response rate of 79% (n = 6,117). Participants were invited for repeated follow-up waves—W3 (1998–2002), W4 (2003–2007), W5 (2008–2012), W6 (2013–2017) and W7 (2018–2022)—with response rates between 75% and 80% from W3 onward. The study design has been described in detail previously (28,29). All participants provided written informed consent at each wave. The study complies with the Declaration of Helsinki and received ethical approval from the Medical Ethics Committees of the Netherlands Organization for Applied Scientific Research and Utrecht University (NL19158.041.07 and NL63779.041.17).

### Eligibility criteria

**Figure 1:**
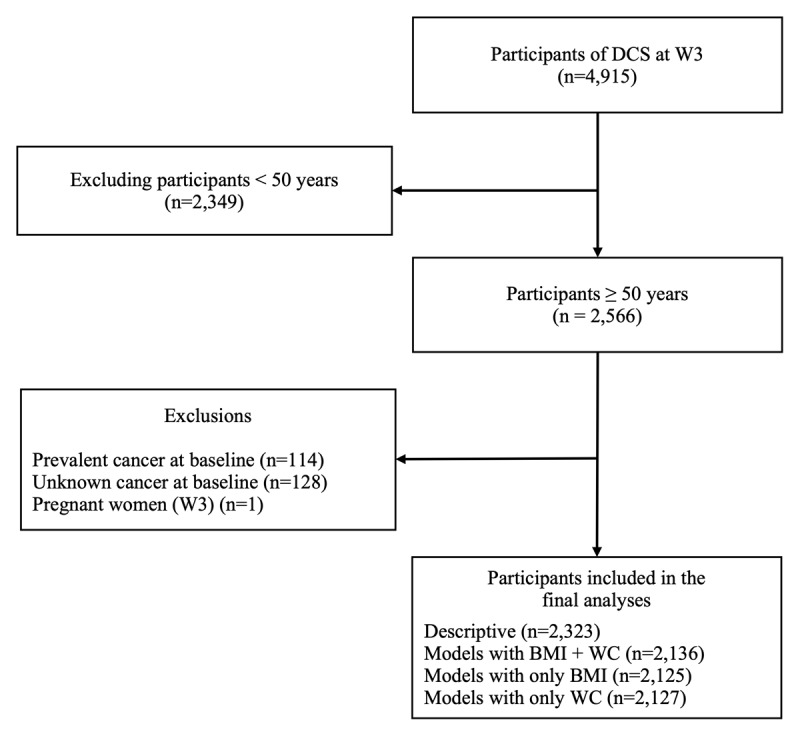
Flowchart of study participant selection.

For the current analysis, we included participants aged 50 years and older at W3 (n = 2,566). We excluded those with a self-report history of cancer at baseline (n = 114), those with unknown cancer status (n = 128), and women who were pregnant at the time of assessment (n = 1). After these exclusions, 2,323 participants were included (Figure 1). For longitudinal modelling analyses, a complete-case approach was applied. Models were fitted using all available person-time contributed by participants with at least one valid measurement of the adiposity exposure of interest during follow-up. Participants without any valid adiposity measurements were excluded from the corresponding models. Missing covariate data resulted in further model-specific exclusions.

### Assessment of adiposity, abdominal obesity and obesity

**Figure 2:**
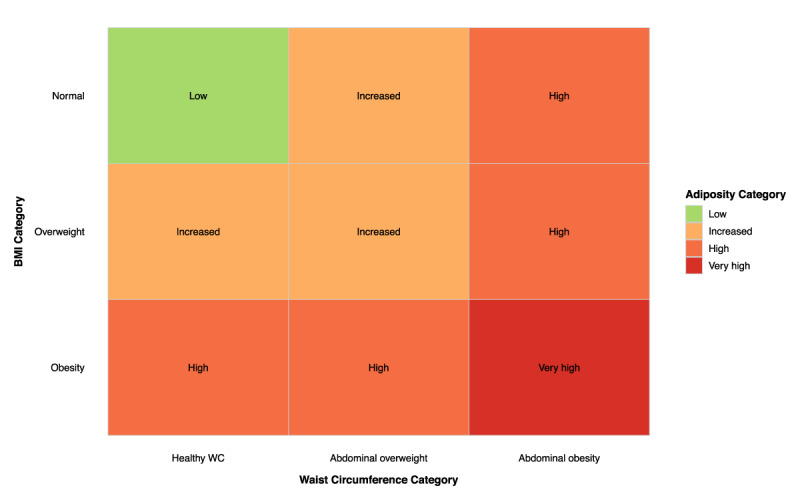
Definition of adiposity categories based on body mass index and waist circumference thresholds. **Legend:** Adiposity categories were defined based on AACE/ ACE guidelines, combining BMI thresholds: normal weight <25 kg/m^2^, overweight 25 to <30 kg/m^2^, and obesity ≥30 kg/m^2^; and WC thresholds: healthy WC <94 cm and <80 cm, abdominal overweight 94≤–<102 cm and 80≤–<88 cm, and abdominal obesity ≥102 cm and ≥88 cm, for men and women, respectively. Abbreviations: BMI, body mass index; WC, waist circumference; AACE, American Association of Clinical Endocrinologists; ACE, American College of Endocrinology.

Anthropometric traits, including height (m), weight (kg), hip and WC (cm), were repeatedly measured in the research centre by trained staff using standard protocols. Adiposity categories were defined based on American Association of Clinical Endocrinologists and the American College of Endocrinology (AACE/ACE) guidelines (30), combining BMI and WC thresholds for abdominal overweight and obesity (80/94 cm and 88/102 cm for women/men) as low, increased, high and very high (Figure 2).

Abdominal overweight and obesity were defined using WC, according to the World Health Organization (WHO) (31), as healthy WC <80cm/<94cm, abdominal overweight 80≤–<88 and 94≤–<102, and abdominal obesity ≥88cm and ≥102 cm for women and men, respectively. BMI was calculated as weight divided by height squared (kg/m^2^) and categorised based on WHO definitions (32): normal weight (<25 kg/m^2^), overweight (25 to <30 kg/m^2^) and obesity (≥30 kg/m^2^).

### Assessment of cardiovascular disease and all-cause mortality

The primary outcome of our study was incident or fatal atherosclerotic CVD, which included definite manifestations of coronary heart disease (angina pectoris, coronary insufficiency, myocardial infarction and coronary death), cerebrovascular disease (stroke, transient ischemic attack, carotid artery stenosis), peripheral arterial disease (claudication or ankle brachial index ≤ 0.90), aortic atherosclerotic disease (abdominal or thoracic aneurysm), heart failure and atrial fibrillation (33). Complete follow-up for mortality and hospital-based morbidity outcomes was achieved through national registry linkage. Vital status was verified using the municipal population register until January 2024 to capture all-cause mortality. Cause of death was ascertained by linkage with Statistics Netherlands, while morbidity data were retrieved through probabilistic linkage with the Dutch Hospital Discharge Registry (34) until December 2023. CVD events, both fatal and non-fatal, were classified according to ICD-9 and ICD-10 codes (Supplementary Table 1) (35,36).

### Covariates

Questionnaires were used to collect data on sex (men and women), household composition (living alone, living with others), education level (low, medium, high), employment status (employed/self-employed, not employed), chronic disease history (e.g., hypertension, diabetes, chronic obstructive pulmonary disease (COPD)), self-perceived health (good/excellent, moderate, poor) and lifestyle behaviours such as physical activity (PA), smoking, alcohol use, diet and sleep. PA was categorised using the Cambridge Physical Activity Index (inactive to active) (37), and cigarettes per day were calculated for smokers. Alcohol consumption was reported as no (never/used to), occasional (<1 glass/week) and yes (with further assessment of glasses/week). Diet was evaluated using a validated 178-item food frequency questionnaire. A modified version of the Dutch Healthy Diet Index 2015 (DHD-15) was calculated, with scores ranging from 1 to 130, with higher scores indicating better adherence to dietary guidelines (38).

Biomedical repeated measurements included blood pressure (BP) (systolic/diastolic, mmHg) and random blood glucose, collected from R1 to R7. Hypertension was defined according to WHO (39) as systolic BP ≥140 mmHg and/or diastolic BP ≥90 mmHg and/or the use of antihypertensive medication. Diabetes mellitus was defined as a random plasma glucose of 11.1 mmol/L (≥200 mg/dl) or self-report. Lipid profiles were measured using enzymatic methods, and hypercholesterolaemia was defined as total cholesterol ≥6.5 mmol/L or the use of lipid-lowering therapy. Low high-density lipoprotein (HDL) cholesterol was defined as <1.03 mmol/L for men and <1.29 mmol/L for women, according to the National Cholesterol Education Program-Adult Treatment Panel III (NCEP-ATPIII) criteria (40).

### Data analysis

Baseline characteristics were summarised as means (SD) or medians (IQR) for continuous variables and as counts (%) for categorical variables. Sex differences were tested using Chi-square tests for categorical variables and Kruskal-Wallis tests for continuous variables. We estimated total LE, CVD-free LE, and LE with CVD by adiposity, WC or BMI categories using a multistate life table approach (41). This method models transitions between three health states: free of CVD, CVD, and death. Transitions included: i) CVD-free to CVD; ii) CVD-free to death; and iii) CVD to death. No backflows were allowed, and only the first event within a state was considered (Supplementary Figure 1).

To evaluate differences in LE across adiposity, WC and BMI categories, we proceeded in three steps. First, we estimated overall sex- and age-specific transition rates for each transition using survival models with a Gompertz distribution. Second, adjusted hazard ratios (HRs) were estimated using Poisson regression with a Gompertz distribution (42), for each non-reference category within each exposure as follows: (i) adiposity (BMI + WC): moderate, high, very high versus low (reference); (ii) WC: abdominal overweight, abdominal obesity versus healthy WC (reference); (iii) BMI: overweight, obesity versus normal weight (reference). Models were adjusted for pre-specified baseline confounders to reduce bias from factors related to both adiposity and the outcomes, including baseline age, household composition, education, PA, diet (DHD-15), smoking (status and cigarettes/day for current smokers), alcohol use, and COPD. All survival models used robust standard errors clustered at the individual level to account for repeated measures.

Third, we calculated sex- and age-specific prevalences of the adiposity/BMI/WC categories (10-year bands), stratified by CVD status. Finally, separated weighted multistate life tables were calculated for each exposure level and sex independently, incorporating each of the three transitions that were estimated separately, considering cohort life table assumptions. The multistate life table started at age 50 years and closed at age 80 years. We derived LE, their differences, and 95% percentile confidence intervals (CIs) via parametric bootstrap resampling (1,000 iterations). The workflow is summarised in Supplementary Figure 1 and follows prior applications of multistate life table methods (17,43,44,45).

To assess the consistency of our primary findings, we conducted four prespecified sensitivity analyses: (i) we repeated all models using WC and BMI separately as the main exposures to minimise collinearity-related instability and facilitate interpretability; (ii) we examined correlations between adiposity indicators descriptively, and sensitivity analyses including joint BMI and WC were performed; (iii) we modelled them as continuous variables using restricted cubic splines to explore non-linear exposure-risk relationships and robustness of associations observed in categorical models; (iv) we excluded participants who experienced CVD events or died within the first two years of follow-up to reduce reverse causation; and (v) we excluded participants with baseline comorbidities to minimise confounding. We evaluated multicollinearity of the covariates using variance inflation factors (VIF) and tolerance from linear models including all covariates; all VIFs were <5 and tolerances >0.20, indicating no concerning multicollinearity. Although VIFs were examined using linear approximations, we recognise their limited applicability to non-linear survival models.

To evaluate whether the Gompertz model provided the best fit to the data, we compared model fit with alternative parametric specifications (Weibull and flexible parametric models; Supplementary Table 3). We used Gompertz proportional hazards models because cardiovascular event rates increase approximately exponentially with attained age, a pattern well captured by the Gompertz hazard. Compared with the Weibull model, which assumes a polynomial time dependency, the Gompertz distribution provides a parsimonious and biologically plausible representation of adult age-related risk (42,46). Formal Schoenfeld-residual-based diagnostics are not directly applicable to parametric Gompertz models. Instead, potential age-dependence of adiposity effects was explored using attained-age stratified Gompertz models (<65 vs ≥65 years), given the limited numbers of events precluding stable estimation of continuous time-varying interactions.

Analyses were performed in Stata 16.0 (StataCorp) and R 4.1.3 (R Foundation for Statistical Computing). Estimates were interpreted based on effect size and precision rather than *p-*values alone. Two-sided *p*-values <0.05 were considered statistically significant.

## Results

### Baseline characteristics of the study population

At baseline, the median age was 57 years, and ~50% were women. High and very high adiposity categories were more prevalent in women than men (high: 39% vs. 24%; very high: 22% vs. 16%). Women were also more likely to have a high prevalence of abdominal obesity than men (60% vs. 40%), while men more often had a higher education level (26%), higher alcohol consumption (75%), and were more frequently former smokers (56.6%). Cardiometabolic comorbidities, including hypertension (49.5%), diabetes (4.8%) and low HDL-cholesterol (14.7%), were more prevalent in men (Table 1).

**Table 1:** Baseline characteristics of the study population.


CHARACTERISTICS	MEN (n = 1,166)	WOMEN (n = 1,157)

*Population*		

Age at baseline, y	57.9 [53.9, 63.7]	57.7 [53.3, 63.2]

Age, 10y		

50–59	689 (59.1)	688 (59.5)

60–69	463 (39.7)	447 (38.6)

70–79	14 (1.2)	22 (1.9)

*Anthropometry*		

Adiposity categories^a^		

Low	208 (17.9)	153 (13.4)

Increased	483 (41.7)	291 (25.5)

High	282 (24.3)	450 (39.4)

Very high	186 (16.0)	247 (21.6)

WC categories^b^		

Healthy WC	308 (26.5)	167 (14.5)

Abdominal overweight	390 (33.6)	286 (24.9)

Abdominal obesity	464 (39.9)	695 (60.5)

BMI categories^c^		

Normal	293 (25.3)	389 (34.1)

Overweight	677 (58.4)	502 (44.0)

Obesity	190 (16.4)	250 (21.9)

*Social and economic status*		

House composition, living with others	1,084 (93.3)	979 (85.0)

Education		

Lower	127 (10.9)	147 (12.8)

Medium	739 (63.6)	821 (74.3)

High	296 (25.5)	184 (16.0)

Employment, employed and self-employed	627 (51.7)	359 (30.7)

*Lifestyle variables*		

Physical activity^d^		

Inactive	166 (14.2)	138 (11.9)

Moderately inactive	338 (29.0)	362 (31.3)

Moderately active	302 (25.9)	342 (29.6)

Active	360 (30.9)	315 (27.2)

Health perception		

Good/excellent	276 (23.7)	279 (24.1)

Intermediate	728 (62.6)	728 (62.9)

Reasonably/poor	159 (13.7)	150 (13.0)

Number of hours of sleep per day		

≤6 hours	230 (19.8)	215 (18.7)

7 hours	454 (39.1)	402 (35.0)

≥8 hours	478 (41.1)	533 (46.3)

Smoking		

Never	266 (22.8)	522 (45.2)

Current	275 (23.6)	250 (21.6)

Former	625 (53.6)	384 (33.2)

Cigarette use in current smokers, no./day	15.0 [10.0, 20.0]	12.0 [8.0, 20.0]

Dutch Healthy Diet index 2015	64.0 [54.3, 72.4]	70.1 [60.8, 78.6]

Alcohol consumption, glasses/week^e^		

No, never/no, used to	101 (8.7)	226 (19.7)

Occasionally, less than 1	154 (13.2)	268 (23.3)

Yes, 1 or 2	38 (3.3)	83 (7.2)

Yes, 3–14; 3–7	514 (44.1)	265 (23.0)

Yes, ≥15; ≥8	359 (30.8)	308 (26.8)

Alcohol consumption in drinkers, glass/week	8.0 [2.0, 16.0]	2.0 [0.0, 8.0]

*Comorbidities and treatment* ^f^		

Hypertension, yes	576 (49.5)	514 (44.8)

Hypertension treatment, yes	165 (19.2)	203 (21.2)

Diabetes, yes	56 (4.8)	41 (3.5)

Diabetes treatment, yes	48 (4.0)	35 (3.0)

Hypercholesterolaemia, yes	256 (22.0)	392 (34.2)

Hypercholesterolaemia treatment, yes	12 (1.3)	12 (1.5)

Low-HDL, yes	171 (14.7)	46 (4.0)

COPD, yes^g^	186 (16.0)	188 (16.2)

*Biomarkers*		

Serum lipids (mmol/L)		

Total cholesterol	5.8 [5.1, 6.4]	6.1 [5.4, 6.8]

HDL	1.2 [1.0, 1.4]	1.5 [1.2, 1.7]


Median [IQR] values for continuous variables and within-category proportions (%) were reported for categorical variables. ^a^Adiposity categories (AACE/ACE guidelines (30)) combining BMI and WC thresholds, as low, moderate, high and very high. ^b^WC categories (WHO (31)); healthy WC <94 cm and <80, abdominal overweight 94≤–<102 cm and 80≤–<88 cm, and abdominal obesity ≥102 cm and ≥88 cm, for men and women, respectively. ^c^BMI categories (WHO (32)); normal weight <25 kg/m^2^, overweight 25 to <30 kg/m^2^, and obesity ≥30 kg/m^2^. ^d^Physical activity based on the Cambridge Physical Activity Index (37). ^e^Sex-specific categories of alcohol consumption in drinkers (glasses/week). In men: yes, 1 or 2 g/wk; yes, 3–14 g/wk; yes, ≥15 g/wk. In women: yes, 1 or 2 g/wk; yes, 3–7 g/wk; yes, ≥7 g/wk. ^f^Treatment for hypertension, diabetes and hypercholesterolaemia were self-reported. ^g^COPD based on self-reported symptoms/complaints: attacks of shortness of breath when walking, almost daily cough (3 months/year), and giving up mucus daily (3 months/year). Abbreviations: IQR, interquartile range; BMI, body mass index; WC, waist circumference; HDL, high-density lipoprotein; COPD, chronic obstructive pulmonary disease.

### Associations between adiposity categories, cardiovascular risk and mortality

**Figure 3:**
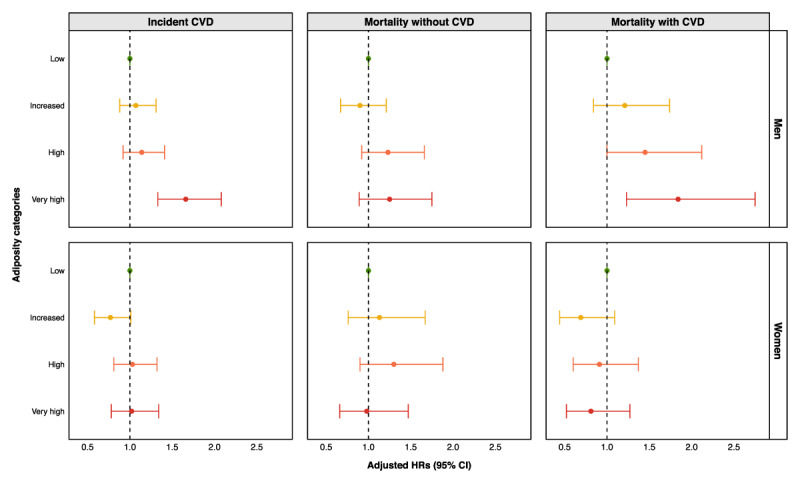
Adjusted HRs for incident CVD and all-cause mortality by adiposity categories, stratified by sex. **Legend:** HRs and 95% CIs for (i) incident CVD and (ii) all-cause mortality among participants with and without CVD, comparing moderate, high and very high versus low category of adiposity. Adiposity categories were defined by combining BMI and WC thresholds: low, moderate, high and very high. Models adjusted for age, household composition, education, physical activity, DHD-15 index, smoking (status and cigarettes/day in current smokers), alcohol consumption and COPD. Abbreviations: BMI, body mass index; CI, confidence interval; COPD, chronic obstructive pulmonary disease; CVD, cardiovascular disease; HRs, hazard ratios; WC, waist circumference.

Over 22 years of follow-up, 695 incident nonfatal CVD events (401 in men and 294 in women) and 587 all-cause deaths (323 in men and 264 in women) occurred. Most associations between adiposity category and CVD morbidity or mortality were not statistically significant (Figure 3; Table 2). Among men, the HR for high (vs. low) risk was 1.14 (95% CI, 0.92 to 1.41), whereas among women, the corresponding HR was 1.03 (95% CI, 0.81 to 1.32). Only men in the very high adiposity category showed clear evidence of an increased risk of CVD, with an HR of 1.66 (95% CI, 1.33 to 2.08) compared to those in the low adiposity category.

**Table 2:** Adjusted HRs for incident CVD and all-cause mortality by adiposity category and sex.


TRANSITION	ADIPOSITY CATEGORY^d^	MEN	WOMEN

		HR (95% CI)^¶^	HR (95% CI)^¶^

Incident CVD^a^	Low	1.0	1.0

	Increased	1.07 (0.88, 1.31)	0.77 (0.58, 1.01)

	High	1.14 (0.92, 1.41)	1.03 (0.81, 1.32)

	Very high	**1.66 (1.33, 2.08)**	1.02 (0.78, 1.34)

Mortality among those without CVD^b^	Low	1.0	1.0

	Increased	0.90 (0.67, 1.21)	1.13 (0.76, 1.67)

	High	1.23 (0.92, 1.66)	1.30 (0.90, 1.88)

	Very high	1.25 (0.89, 1.75)	0.98 (0.66, 1.47)

Mortality among those with CVD^c^	Low	1.0	1.0

	Increased	1.21 (0.84, 1.74)	0.69 (0.44, 1.09)

	High	1.45 (1.00, 2.12)	0.91 (0.60, 1.37)

	Very high	**1.84 (1.23, 2.75)**	0.81 (0.53, 1.27)


^a^Calculations made with 1,044 men and 1,092 women. ^b^Calculations made with 1,044 men and 1,092 women. ^c^Calculations made with 1,044 men and 1,092 women. ^d^Adiposity categories (AACE/ACE guidelines), combining BMI and WC thresholds: low, increased, high and very high. ^¶^Models adjusted for age, household composition, education, physical activity, DHD-15 index, smoking (status and cigarettes/day in current smokers), alcohol consumption and COPD. Significant associations in bold. Abbreviations: BMI, body mass index; CI, confidence interval; CVD, cardiovascular disease; HR, hazard ratio; WC, waist circumference.

For all-cause mortality, patterns differed by sex and CVD status, although most associations did not reach statistical significance (Figure 3; Table 2). Among women without CVD, HRs were 1.30 (95% CI, 0.90 to 1.88) for high and 0.98 (95% CI, 0.66 to 1.47) for very high adiposity categories, compared with the low category. In men without CVD, the HR for the very high adiposity category was 1.23 (95% CI, 0.92 to 1.66). Among participants with CVD, the HRs for the high versus low adiposity category were 1.45 (95% CI, 1.00 to 2.12) in men and 0.91 (95% CI, 0.60 to 1.37) in women, with CIs compatible with both increased and decreased risks. Only men in the very high adiposity category showed clear evidence of an increased risk of mortality, with an HR of 1.84 (95% CI, 1.23 to 2.75) compared to those in the low adiposity category.

Across age-stratified Gompertz models, associations between adiposity and disease transitions varied by age and sex, but estimates were imprecise due to limited numbers of events. Very high adiposity was associated with higher risks primarily among men aged <65 years, whereas associations were largely absent at older ages and among women (data not shown).

### Total LE and LE with and without CVD

**Figure 4:**
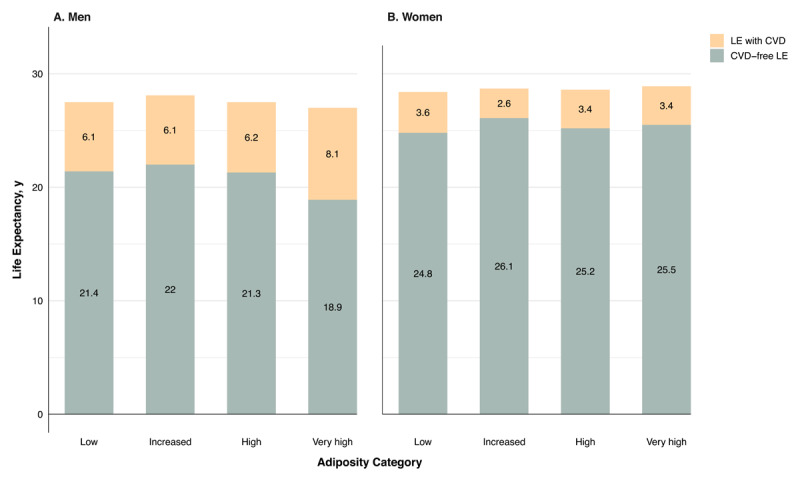
Effect of adiposity on sex-specific LE with and without CVD at age 50 years. **Legend:** Total LE at age 50 divided into CVD-free LE and LE with CVD, by sex and adiposity categories (low, increased, high, very high) defined by combining BMI and WC thresholds. Abbreviations: BMI, body mass index; CVD, cardiovascular disease; LE, life expectancy; WC, waist circumference.

Estimates of total LE, CVD-free LE, and LE with CVD by sex and adiposity category are shown in Figure 4 and Supplementary Table 4. At age 50, total LE did not differ significantly across adiposity categories in either sex. Compared with men in the low adiposity category, those in the very high category lived 2.6 years less free of CVD (difference: –2.6 years; 95% CI, –3.6 to –1.5), and two years more with CVD (95% CI, 0.9 to 2.9). Men in the increased or high adiposity categories had small, not statistically significant differences in years lived with CVD compared with the low category (Figure 4; Supplementary Table 3).

Among women, associations between adiposity and LE outcomes were not statistically significant. Point estimates for higher adiposity categories were slightly higher for CVD-free LE and slightly lower for years lived with CVD; however, CIs included the null and were compatible with both beneficial and adverse differences. For example, women in the very high adiposity category lived 0.6 years more free of CVD (difference: 0.6 years; 95% CI, –0.1 to 1.4), and 0.2 years less with CVD (difference: –0.2; 95% CI, –1.0 to 0.4) compared with the low category. Similar patterns were observed for the high adiposity category, with small and imprecisely estimated differences in years lived free of CVD and with CVD (Supplementary Table 4).

### Sensitivity analyses

Among the participants, approximately 10% of men and 6% of women were excluded from survival models due to missing time-varying exposures. Moreover, given the conceptual nature of exposure trajectories and the complexity of multistate survival models, multiple imputation was not applied. Instead, the potential impact of exclusions was assessed through descriptive comparisons stratified by sex. Differences in age, adiposity measures and lifestyle factors were minor and unlikely to materially bias the results.

Given the strong correlation between BMI and WC (*r* = 0.81, p = 0.00), these adiposity measures were evaluated in separate models (Supplementary Table 2), and they showed broadly consistent directions and magnitudes of association with incident CVD and mortality for both sexes, despite shifts in classification between WC and BMI categories (Supplementary Figure 4). Mutual adjustment for BMI and WC resulted in attenuation of associations and increased uncertainty, consistent with collinearity between measures of general and central adiposity.

Restricted cubic spline analyses demonstrated statistically significant non-linear associations between both BMI and WC and incidence of CVD (tests for non-linearity p < 0.05). Risk increased at higher levels of adiposity, while estimates at the lower and upper extremes were imprecise, as reflected by wide CIs. Overall, spline-based estimates were consistent with the categorical analyses and did not indicate materially different risk patterns (Supplementary Figures 5 and 6).

When using BMI, LE outcomes followed similar patterns to those observed with WC, although estimated differences in years lived free of CVD and with CVD were larger for BMI than for WC, particularly in men. For example, compared with men of normal weight, men with obesity (BMI-based) lived 1.3 years less free of CVD (difference: –1.3 years; 95% CI, –2.3 to –0.2). In contrast, compared with men with a healthy WC, men with abdominal obesity lived 0.6 years less free of CVD (95% CI, –1.6 to 0.5), with CIs for WC-based estimates spanning the null.

Among women, no statistically significant differences in years lived with or without CVD were observed between those with obesity and those of normal weight, consistent with WC-based results (Supplementary Table 4). Findings for total LE, CVD-free LE, and LE with CVD were similar after excluding participants with baseline comorbidities and those who experienced a CVD event or died within the first two years of follow-up (data not shown).

## Discussion

In this population-based cohort of middle-aged and older adults followed for over two decades, adiposity was not associated with differences in total LE at age 50. However, this apparent neutrality in total lifespan masked meaningful differences in the distribution of life years with and without CVD. In particular, men in the very high adiposity category experienced earlier onset of CVD and a clear expansion of morbidity, characterised by fewer years lived free of CVD and substantially more years lived with CVD compared with men in the low adiposity category. Consistent with this pattern, only men in the very high adiposity category had a higher risk of incident CVD (HR 1.66; 95% CI, 1.33 to 2.08). By contrast, among women, adiposity was not associated with clear differences in any LE outcomes. By using a multistate cohort life table approach with age-specific transition hazards, we account for competing risks and capture the joint dynamics of adiposity, CVD onset, and mortality across the life course, thereby providing a more realistic representation of long-term health trajectories than single-endpoint analyses.

Current guidelines recommend assessing adiposity with both BMI and WC in older adults, given age-related multimorbidity, body composition shifts, and sarcopenia, to refine cardiometabolic risk stratification and better characterise mortality and LE (22,23,24). Using composite adiposity categories, our findings showed clearer differences in LE-specific outcomes, particularly in men. Men with very high adiposity (WC + BMI), abdominal obesity (WC), or obesity (BMI) experienced earlier CVD onset and lived roughly 1–2 additional years with CVD compared to men with low adiposity/healthy WC/normal weight, indicating an expansion of morbidity rather than compression of disease into a shorter period at the end of life. Although the observed effect sizes were modest, our findings are broadly consistent with the Tromsø, ESTHER, Rotterdam, and Framingham Heart Study cohorts, which reported similar patterns, albeit with generally larger effect estimates (13,16,17). In contrast, the Research Centre for Prevention and Health study reported fewer years lived with CVD among overweight and obese men, highlighting heterogeneity across cohorts. Among women, we observed no significant associations between adiposity (composite, WC, or BMI) and LE outcomes, in contrast to cohorts reporting up to two additional years lived with CVD among women with overweight/obesity (13,16,17,19).

Several factors may explain these differences. Earlier work relied primarily on BMI, whereas we evaluated WC and a composite indicator (WC + BMI) to better capture central adiposity. Visceral adiposity is more strongly linked to cardiometabolic risk and mortality than general adiposity, and may more accurately reflect obesity-related disease burden in ageing populations (23,24,47). In our sensitivity analyses, however, BMI-based associations with LE were nearly twice as large as WC-based estimates, particularly in men. This suggests that general adiposity may capture a broader morbidity burden extending beyond CVD alone, whereas WC, though more specific to visceral fat and incident CVD, may be attenuated by age-related changes in body shape. Using both measures reduces misclassification (e.g., normal-BMI/high-WC phenotypes) and, as shown by the composite categories, identifies the risk gradient in men (higher incident CVD and more years with CVD in the very-high group). Despite extensive work on BMI or WC separately, few studies have evaluated their joint effects on mortality, and rarely on CVD or LE (51), complicating direct comparisons with our findings. Replication of our findings in future studies is needed to substantiate the added value of using a combined measure of BMI and WC in studying the association between adiposity and CVD.

Our findings highlighted a sex-specific pattern: among participants with CVD, men in the very high versus low adiposity category had an HR 1.84 (95% CI, 1.23 to 2.75), with an opposite effect in women. These results contrast with the Rotterdam Study (17), the FHS (13), and the Tehran Lipid and Glucose Study (TLGS) (19), which reported lower mortality among men with obesity and CVD, and the FHS observations of higher mortality among women with obesity. Biological and socio-behavioural sex differences likely contribute to the divergent LE/mortality and CVD trajectories we observed. Men had a higher baseline prevalence of cardiometabolic risk factors (hypertension, diabetes, smoking and low HDL-cholesterol), which may have amplified the adverse effects of abdominal obesity on disease onset, survival and more years lived with CVD. Furthermore, men accumulate significantly more visceral adipose tissue (VAT) than women, even at the same BMI or WC. VAT is metabolically active and pro-inflammatory (e.g., IL-6, TNF-α, CRP), promoting insulin resistance, endothelial dysfunction, and atherogenesis. Women tend to store more subcutaneous adipose tissue (SAT), which is less metabolically active. This distribution may protect women from the harmful effects of central obesity. However, this sex difference diminishes with ageing and menopause, as oestrogen decline is linked to SAT dysfunction, increased VAT accumulation, and higher cardiometabolic risk in women (24,48,49,50,51). These biological differences, combined with men’s greater baseline risk burden, likely explain the more pronounced reductions in CVD-free LE and longer time lived with CVD observed in men.

In addition, differences in age structure and calendar time may further explain discrepancies with earlier studies. Our cohort, recruited in the late 1990s and followed into the 2020s, reflects intensified prevention and treatment (better hypertension/lipid control and lower smoking), which may attenuate the observed impact of obesity on mortality and delay the onset of CVD. Compared with the Rotterdam Study (17), our participants were younger, more active, better educated (especially women) and had healthier lifestyle profiles—differences that could weaken observed associations with long-term outcomes. Methodological factors (analytic approaches, covariate sets, LE modelling, endpoint definitions) further vary across studies (24,52); nonetheless, sensitivity analyses excluding early events and baseline comorbidities yielded consistent results, supporting robustness.

Strengths of our study include long follow-up, the standardised exposure and outcome assessment, sex-specific modelling of LE outcomes, and an a priori composite categorisation intended to capture both general and central adiposity. Limitations include reliance on baseline measures and time-varying measurement gaps (risk of non-differential misclassification toward the null), limited precision in sex-stratified subgroups, and a predominantly White European cohort, which may limit generalisability. Furthermore, the primary endpoint combined heterogeneous cardiovascular outcomes, but limited numbers of heart failure and atrial fibrillation events precluded adequately powered sensitivity analyses. Spline analyses confirmed non-linear associations for BMI and WC but did not materially alter conclusions from categorical models. Given imprecision at distributional extremes, limited events in stratified analyses, and the clinical relevance of established cut-points, categorical exposures were retained as primary, with splines used for sensitivity analyses. To reduce misclassification in older adults, studies should test age-appropriate WC and composite cut-points and use repeated anthropometry to capture fat redistribution. In parallel, whether parsimonious panels of inflammatory/metabolic biomarkers (e.g., CRP, IL-6, insulin-resistance indices, lipid profiles) improve prediction beyond BMI + WC and whether these biomarkers mediate or modify associations should be evaluated; such markers may aid earlier detection of subclinical disease and prediction of accelerated progression, especially in older adults with accumulated risks (24,48,49,50). Pre-specified assessments of effect modification (e.g., smoking), together with replication in more diverse cohorts, will support generalisability and clinical uptake.

## Conclusions

Very high adiposity at age 50 was not associated with reduced total LE in either sex. However, in men, it was associated with a marked expansion of morbidity, characterised by substantially fewer CVD-free years and longer time lived with CVD. From clinical and public health perspectives, these findings underscore the importance of early, sex-specific interventions to prevent obesity-related CVD. Midlife represents a key window to delay disease onset, reduce years lived with CVD, and promote healthier ageing. Integrating both general and central adiposity measures into routine risk assessments could enhance identification of high-risk individuals and improve the targeting of preventive strategies tailored to sex, age and metabolic profile. Nevertheless, as this is one of the first to use a combination of BMI and WC to measure adiposity and study the association of LE in adults, with and without CVD, future studies are needed to replicate our findings.

## Additional File

The additional file for this article can be found as follows:

10.5334/gh.1558.s1 Supplementary Material. Supplementary Tables 1 to 4 and Figures 1 to 6.

## Data Availability

Data and codebooks supporting this manuscript are available from the corresponding author upon reasonable request, subject to a signed data access agreement.
