# Supplementary material for: Adiposity in Adults and Life Expectancy with and without Cardiovascular Disease. The Doetinchem Cohort Study
Source: Glob Heart. 2026 May 26;21(1):43. doi: 10.5334/gh.1558 (PMC13220739; doi:10.5334/gh.1558)
Supplement: Supplementary Material. — Supplementary Tables 1 to 4 and Figures 1 to 6. [file gh-21-1-1558-s1.pdf]

Supplementary material

Adiposity in adults and life expectancy with and without cardiovascular disease. The Doetinchem Cohort Study

Silvia Juliana Trujillo-Cáceres[a,b] (ORCID: 0000-0002-7626-3822), Annelot P Smit[b,c] (ORCID: 0009-0003-0058-398X), Vicente Artola Arita[a] (ORCID: 0000-0002-3043-1675), Marilyne Menassa[a] (ORCID: 0000-0002-3277-4313), H Susan J Picavet[b] (ORCID: 0000-0002-6895-165X), Klodian Dhana[d] (ORCID: 0000-0002-6397-7009), Oscar H Franco[a] (ORCID: 0000-0002-4606-4929), W M Monique Verschuren[a,b] (ORCID: 0000-0003-2134-4227)

[a] Department of Global Public Health and Bioethics, Julius Center for Health Sciences and Primary Care, University Medical Center (UMC) Utrecht, Utrecht, the Netherlands

[b] Center for Prevention, Lifestyle and Health, National Institute for Public Health and the Environment (RIVM), Bilthoven, The Netherlands

[c] Julius Center for Health Sciences and Primary Care, University Medical Center Utrecht, Utrecht, The Netherlands

[d] Department of Internal Medicine, Division of Epidemiology, Rush Institute of Healthy Aging, Rush University Medical Center, Chicago, Illinois, USA

List of supplementary tables

Supplementary Table 1. Definition of atherosclerotic CVD using ICD-9 and ICD-10 codes

Supplementary Table 2. Adjusted hazard ratios for incident CVD and all-cause mortality by exposure category (BMI and WC) and sex

Supplementary Table 3. Model fit comparison (only final models of adiposity for each transition)

Supplementary Table 4. Total LE, CVD-free LE, and LE with CVD at age 50 and absolute differences, by exposure category (adiposity, WC, BMI) and sex (95% percentile CI)

List of supplementary figures and figure legends

Supplementary Figure 1. Schematic of three health-state transitions and overview of the multistate life-table method

Supplementary Figure 2. Effect of WC categories on sex-specific life expectancy with and without CVD at age 50 years

Supplementary Figure 3. Effect of BMI categories on sex-specific life expectancy with and without CVD at age 50 years

Supplementary Figure 4. Weight category concordance by WC and BMI at baseline, stratified by sex

Supplementary Figure 5. Adjusted hazard ratios for the incidence of cardiovascular disease across waist circumference

Supplementary Figure 6. Adjusted hazard ratios for the incidence of cardiovascular disease across body mass index

Supplementary Table 1. Definition of atherosclerotic CVD using ICD-9 and ICD-10 codes

Entity	ICD-9 Code	Description	ICD-10 Code	Description
Coronary heart disease (CHD)	410	Acute myocardial infarction	I21	Acute myocardial infarction
			I22	Subsequent ST elevation (STEMI) and non-ST elevation (NSTEMI) myocardial infarction
	412	Old myocardial infarction	I23	Certain current complications following ST elevation (STEMI) and non-ST elevation (NSTEMI) myocardial infarction (within the 28-day period)
	411	Other acute and subacute forms of CHD	I24	Other acute CHD
	413	Angina pectoris	I20	Angina pectoris
	414	Other forms of chronic CHD	I25	Chronic CHD
	415.1	Pulmonary embolism and infarction	I26	Pulmonary embolism
Cardiac dysrhythmias	427.3	Atrial fibrillation and flutter	I48	Atrial fibrillation and flutter
	427.5	Cardiac arrest	I46.2	Cardiac arrest due to underlying cardiac condition
			I46.8	Cardiac arrest due to other underlying condition
			I46.9	Cardiac arrest, cause unspecified
Heart failure	428	Heart failure	I50	Heart failure
Cerebrovascular disease	430	Subarachnoid haemorrhage	I60	Nontraumatic subarachnoid hemorrhage
	431	Intracerebral haemorrhage	I61	Nontraumatic intracerebral hemorrhage
	432	Other and unspecified intracranial haemorrhage	I62	Other and unspecified nontraumatic intracranial haemorrhage
	433	Occlusion and stenosis of precerebral arteries	I65	Occlusion and stenosis of precerebral arteries, not resulting in cerebral infarction
	434	Occlusion of cerebral arteries	I66	Occlusion and stenosis of cerebral arteries, not resulting in cerebral infarction
	435	Transient cerebral ischemia	I63	Cerebral infarction
	436	Acute, but ill-defined, cerebrovascular disease	I67	Other cerebrovascular diseases
	437	Other and ill-defined cerebrovascular disease	I68	Cerebrovascular disorders in diseases classified elsewhere
	438	Late effects of cerebrovascular disease	I69	Sequelae of cerebrovascular disease
Diseases of arteries, arterioles and capillaries	440	Atherosclerosis	I70	Atherosclerosis
	441	Aortic aneurysm and dissection	I71	Aortic aneurysm and dissection
	442	Other aneurysm	I72	Other aneurysm
	443.9	Peripheral vascular disease, unspecified	I73.9	Other peripheral vascular diseases
	444	Arterial embolism and thrombosis	I74	Embolism and thrombosis of thoracic aorta
Sudden death cause unknown (ICD- 9) or ill-defined and unknown cause of mortality (ICD-10)	798.2	Death occurring in less than 24 hours from onset of symptoms, not otherwise explained	R99	Ill-defined and unknown cause of mortality
	798.9	Unattended death		

Supplementary Table 2. Adjusted hazard ratios for incident CVD and all-cause mortality by exposure category (BMI and WC) and sex

Transition	Exposure category	Men	Women
		HR (95% CI)[¶]	HR (95% CI)[¶]
WC[a]			
Incident CVD[b]	Healthy	1.0	1.0
	Overweight	1.06 (0.80, 1.39)	0.95 (0.63, 1.41)
	Abdominal obesity	1.23 (0.95, 1.61)	1.06 (0.74, 1.52)
Mortality among those without CVD[c]	Healthy	1.0	1.0
	Overweight	0.94 (0.62, 1.44)	1.09 (0.59, 1.98)
	Abdominal obesity	1.23 (0.82, 1.84)	1.67 (0.99, 2.84)
Mortality among those with CVD[d]	Healthy	1.0	1.0
	Overweight	1.00 (0.62, 1.62)	0.84 (0.43, 1.64)
	Abdominal obesity	1.52 (0.98, 2.35)	1.24 (0.69, 2.21)
BMI[e]			
Incident CVD[f]	Normal weight	1.0	1.0
	Overweight	1.04 (0.81, 1.33)	1.05 (0.79, 1.38)
	Obesity	**1.42 (1.04, 1.94)**	1.04 (0.75, 1.44)
Mortality among those without CVD[g]	Normal weight	1.0	1.0
	Overweight	0.93 (0.64, 1.35)	1.21 (0.83, 1.77)
	Obesity	1.08 (0.66, 1.77)	1.18 (0.73, 1.89)
Mortality among those with CVD[h]	Normal weight	1.0	1.0
	Overweight	1.01 (0.67, 1.53)	0.94 (0.69, 1.28)
	Obesity	1.45 (0.88, 2.39)	1.25 (0.87, 1.79)

[a] WC categories (WHO): healthy WC <80 and <94cm, abdominal overweight 80≤WC<88cm and 94≤WC<102cm, and abdominal obesity ≥88 and ≥102cm, for women and men, respectively.

[b] Calculations made with 1,011 men and 1,054 women.

[c] Calculations made with 1,011 men and 1,054 women.

[d] Calculations made with 1,040 men and 1,087 women.

[e] BMI categories (WHO): normal weight: <25 kg/m²; overweight: 25<30 kg/m²; obesity: ≥30 kg/m².

[f] Calculations made with 1,008 men and 1,048 women.

[g] Calculations made with 1,008 men and 1,048 women.

[h] Calculations made with 1,041 men and 1,084 women.

[¶] Models adjusted for age, household composition, education, physical activity, DHD-15 index, smoking (status and cigarettes/day in current smokers), alcohol consumption, and COPD. Significant associations in bold.

Abbreviations: CI, confidence interval; CVD, cardiovascular disease; HR, hazard ratio; WC, waist circumference

Supplementary Table 3. Model fit comparison (only final models of adiposity for each transition)

Transition and model	Men		Women	
	**AIC**	**BIC**	**AIC**	**BIC**
Incident CVD				
Gompertz	675.30	709.34	1115.49	1150.38
Weibull	673.10	707.14	1111.43	1146.31
Flexible parametric (df=4)	12468.49	12488.91	11100.62	11177.36
Mortality among those without CVD				
Gompertz	696.63	730.67	798.62	833.50
Weibull	703.11	737.15	800.99	835.88
Flexible parametric (df=4)	689.76	764.64	776.53	853.28
Mortality among those with CVD				
Gompertz	817.19	851.23	852.40	887.28
Weibull	815.26	849.30	849.99	884.87
Flexible parametric (df=4)	822.97	897.85	835.25	911.99

Models were adjusted for covariates as described in the Methods.

Supplementary Table 4. Total LE, CVD-free LE, and LE with CVD at age 50 and absolute differences, by exposure category (adiposity, WC, BMI) and sex (95% percentile CI)

**Exposure categories**	**Total LE (y)**	**Differences in total LE (y)**	**CVD-free LE (y)**	**Differences in CVD-free LE (y)**	**LE with CVD (y)**	**Differences in LE with CVD (y)**
Men						
Adiposity[¥]						
Low	27.5 (27.0, 28)	Ref.	21.4 (20.4, 22.4)	Ref.	6.1 (5.3, 7.1)	Ref.
Moderate	28.1 (28.0, 28,2)	0.6 (0.0, 1.2)	22.0 (21.8, 22.2)	0.6 (-0.4, 1.7)	6.1 (5.9, 6.3)	0.0 (-1.1, 0.9)
High	27.5 (27.4, 27.6)	0.0 (-0,5, 0.6)	21.3 (21.0, 21.5)	-0.1 (-1.1, 1.0)	6.2 (6.0, 6.4)	0.1 (-0.9, 1.0)
Very high	27.0 (26.8, 27.1)	-0.6 (-1.1, 0.1)	18.9 (18.5, 19.2)	**-2.6 (-3.6, -1.5)**	8.1 (7.8, 8.4)	**2.0 (0.9, 2.9)**
WC categories[¶]						
Healthy WC	27.7 (27.2, 28.1)	Ref.	21.5 (20.5, 22.4)	Ref.	6.1 (5.3, 7.1)	Ref.
Abdominal overweight	28,1 (28,0, 28,2)	0.43 (-0.02, 1.06)	22.3 (22.1, 22.5)	0.80 (-0.16, 1.89)	5.8 (5,6, 6.0)	-0.37 (-1.39, 0.51)
Abdominal obesity	27.6 (27.5, 27.7)	-0.14 (-0.63, 0.50)	20.7 (20.5, 20.9)	-0.79 (-1.77, 0.31)	6.8 (6.6, 7.0)	0.65 (-0.39, 1.58)
BMI categories[±]						
Normal weight	27.5 (27.0, 28.0)	Ref.	21.4 (20.3, 22.3)	Ref.	6.2 (5.3, 7.2)	Ref.
Overweight	27.9 (27.8, 28.0)	0.4 (-0.1, 1.1)	22.5 (22.3, 22.6)	**1.1 (0.1, 2.2)**	5.5 (5.3, 5.6)	-0.7 (-1.7, 0.2)
Obesity	27.4 (27.3, 27.5)	-0.1 (-0.6, 0.6)	19.8 (19.5, 20.1)	**-1.5 (-2.5, -0.4)**	7.6 (7.4, 7.9)	**1.4 (0.4, 2.4)**
Women						
Adiposity[¥]						
Low	28.5 (28.0, 28.8)	Ref.	24.8 (24.1, 25.5)	Ref.	3.6 (3.0, 4.4)	Ref.
Moderate	28.8 (28.7, 28.9)	0.3 (-0.1, 0.8)	26.1 (26.0, 26.3)	**1.3 (0.5, 2.1)**	2.6 (2.5, 2.8)	**-1.0 (-1.8, -0.4)**

High	28.6 (28.5, 28.7)	0.1 (-0.3, 0.6)	25.2 (25.0, 25.3)	0.3 (-0.4, 1.1)	3.4 (3.3, 3.6)	-0.2 (-1.0, 0.4)
Very high	28.9 (28.8, 29.0)	0.4 (0.0, 0.8)	25.5 (25.3, 25.6)	0.6 (-0.1, 1.4)	3.4 (3.3, 3.6)	-0.2 (-1.0, 0.4)
WC categories¶						
Healthy WC	28.6 (28.2, 29.0)	Ref.	24.9 (24.0, 25.5)	Ref.	3.8 (3.1, 4.5)	Ref.
Abdominal overweight	28.9 (28.8, 29.0)	0.3 (-0.1, 0.7)	26.0 (25.8, 26.1)	**1.1 (0.3, 1.9)**	2.9 (2.8, 3.1)	**-0.9 (-1.6, -0.2)**
Abdominal obesity	28.8 (28,7, 28.9)	0.2 (-0.1, 0.6)	25.2 (25.0, 25.3)	0.3 (-0.5, 1.1)	3.7 (3.5, 3.8)	-0.1 (-0.8, 0.6)
BMI categories±						
Normal weight	28.3 (27.8, 28.7)	Ref.	24.6 (23.8, 25.3)	Ref.	3.7 (3.1, 4.4)	Ref.
Overweight	28.7 (28.6, 28.8)	0.4 (0.0, 0.9)	24.7 (24.6, 24.8)	0.1 (-0.7, 0.9)	4.0 (3.8, 4.1)	0,2 (-0.5, 0.9)
Obesity	28.9 (28.8, 29.0)	**0.6 (0.2, 1.2)**	24.9 (24.7, 25.0)	0.3 (-0.5, 1.1)	4.0 (3.9, 4.2)	0.3 (-0.4, 1.0)

¥Adiposity categories (AACE/ACE guidelines), combining BMI and WC thresholds as low, moderate, high risk, and very high.

¶ WC categories (WHO): healthy WC <80 and <94cm, abdominal overweight 80≤WC<88cm and 94≤WC<102cm, and abdominal obesity ≥88 and ≥102cm, for women and men, respectively.

± BMI categories (WHO): normal weight: <25 kg/m²; overweight: 25<30 kg/m²; obesity: ≥30 kg/m².

Significant associations in bold.

Abbreviations: BMI, body mass index; CI, confidence interval; CVD, cardiovascular disease; LE, life expectancy; WC, waist circumference.

Supplementary Figure 1. **Schematic of three health-state transitions and overview of the multistate life-table method**

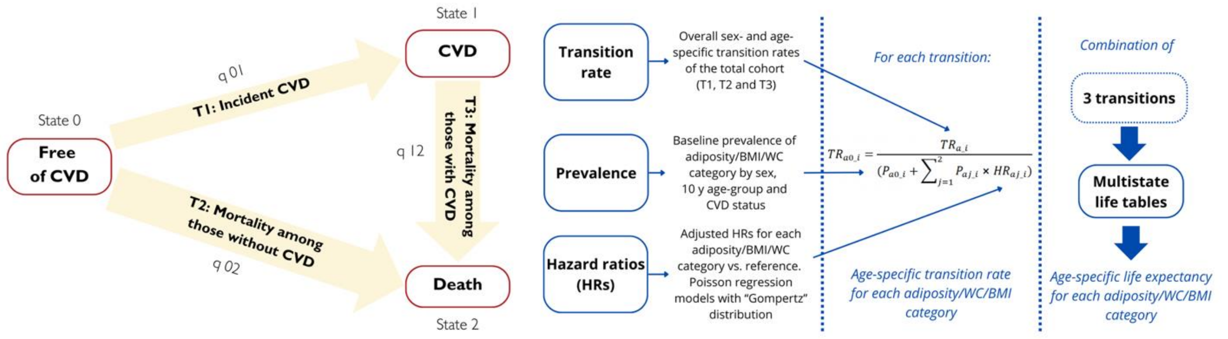

Left: three states, (0) CVD-free, (1) CVD, (2) death, and three transitions q01 (CVD-free→CVD), q02 (CVD-free→death), q12 (CVD→death). No backflows were allowed, and only the first event was considered. Right: overview of the multistate life-table approach used to estimate differences in LE by adiposity/weight categories. Weighted transition rates were obtained by: (1) estimating sex- and age-specific transition hazards using Gompertz survival models; (2) estimating adjusted HRs comparing non-reference categories within each exposure, adiposity (BMI+WC: increased/high/very high vs low), WC (abdominal overweight/abdominal obesity vs healthy WC), BMI (overweight/obesity vs normal weight), with adjustment for confounders; and (3) calculating baseline prevalences of exposure categories by sex, 10-year age bands, and CVD status. Separate life tables were created for each sex and exposure; the life table started at age 50 and ended at age 80.

Abbreviations: CVD, cardiovascular disease; HR, hazard ratio; TR, transition rate.

Supplementary Figure 2. Effect of WC category on sex-specific life expectancy with and without CVD at age 50 years

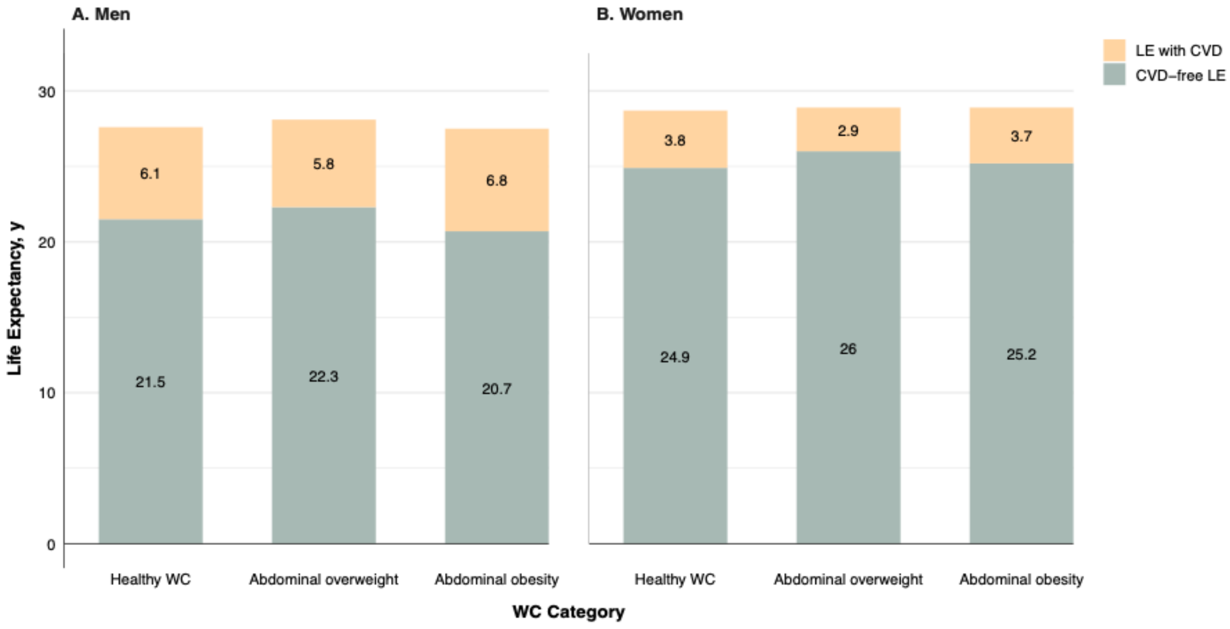

Total LE at age 50, divided into CVD-free LE and LE with CVD, by sex and WC category (WHO): women, healthy WC <80cm; abdominal overweight 80≤WC<88cm; abdominal obesity ≥88 cm; men, healthy WC <94cm; abdominal overweight 94≤WC<102cm; abdominal obesity ≥102cm.

Abbreviations: CVD, cardiovascular disease; LE, life expectancy; WC, waist circumference

Supplementary Figure 3. Effect of BMI category on sex-specific life expectancy with and without CVD at age 50 years

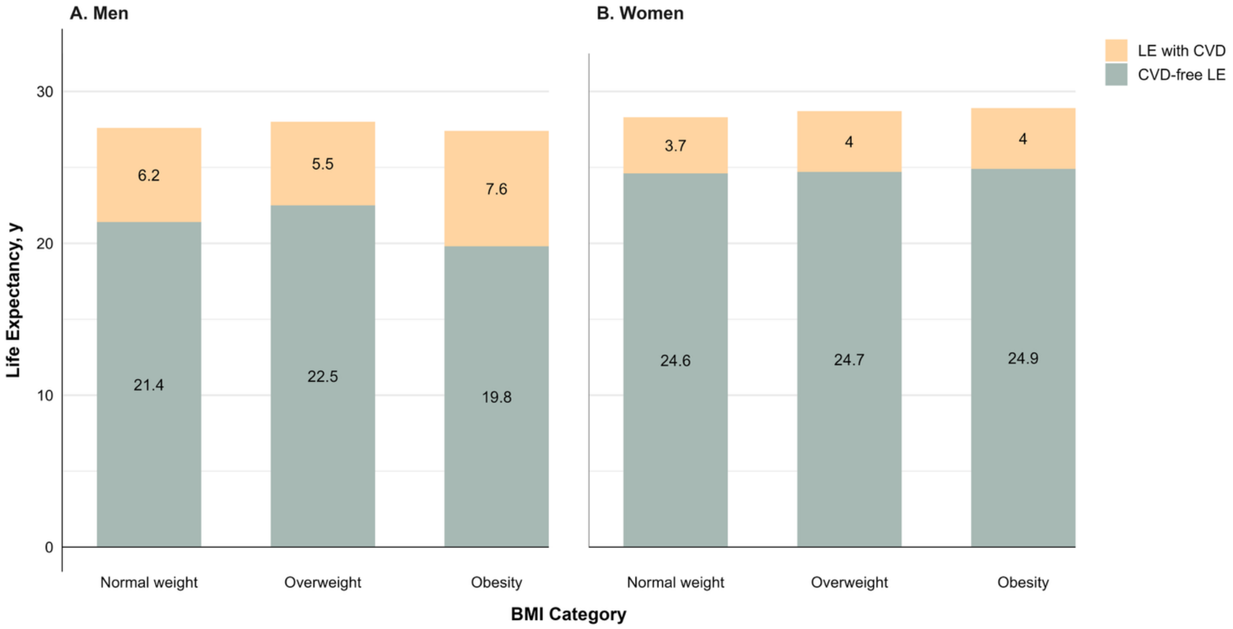

Total LE at age 50, divided into CVD-free LE and LE with CVD, by sex and BMI category (WHO): normal weight <25 kg/m²; overweight 25-<30 kg/m²; obesity ≥30 kg/m².

Abbreviations: BMI, body mass index; CVD, cardiovascular disease; LE, life expectancy.

Supplementary Figure 4. Concordance between WC and BMI-based categories at baseline, stratified by sex

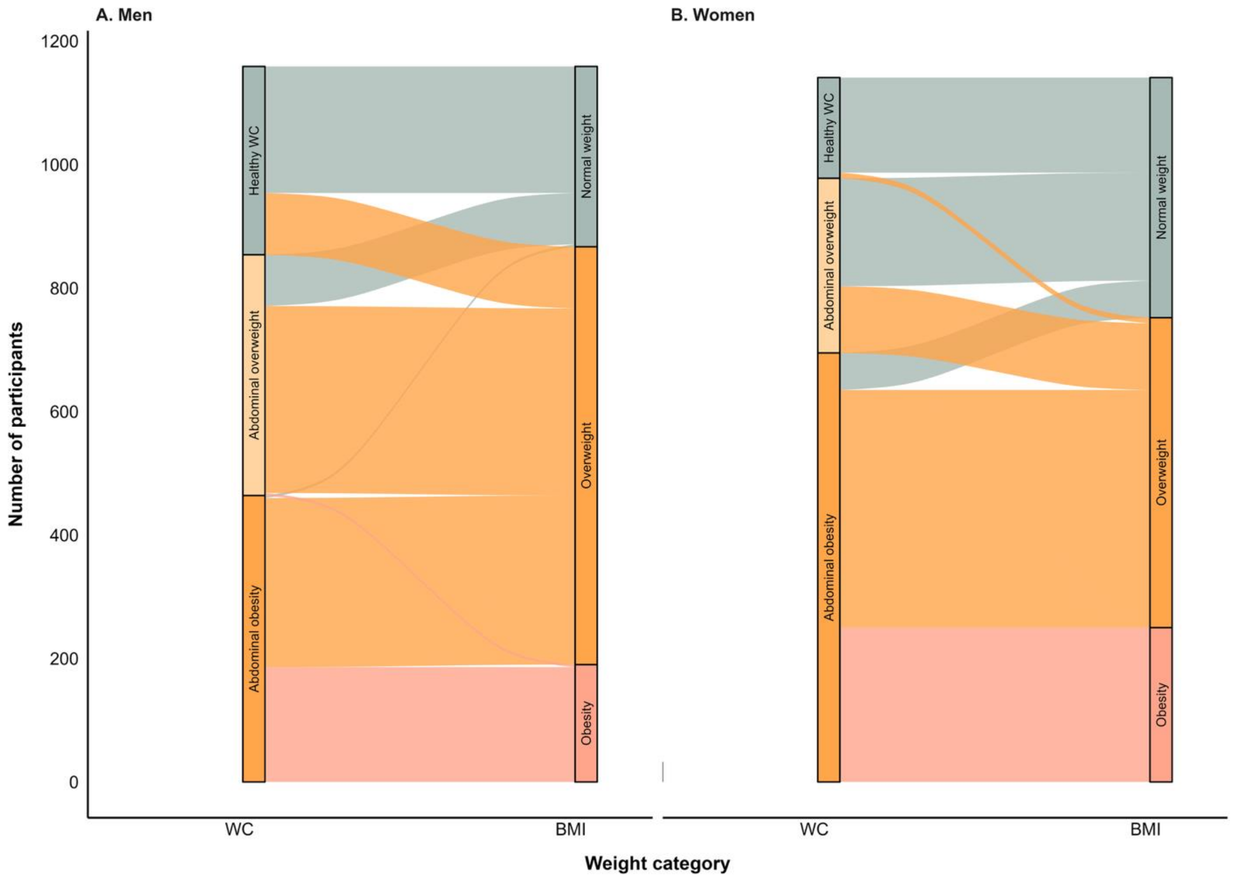

Alluvial plots show participant flow between WC- and BMI-based categories at baseline by sex. WC thresholds: men, healthy <94 cm; abdominal obesity ≥102 cm; women, healthy <80 cm; abdominal obesity ≥88 cm. BMI categories per WHO. While many participants mapped to corresponding categories across measures, a substantial proportion classified as abdominally obese by WC were overweight by BMI, highlighting differences between general and central adiposity.

Abbreviations: BMI, body mass index; WC, waist circumference.

Supplementary Figure 5. Adjusted hazard ratios for the incidence of cardiovascular disease across waist circumference

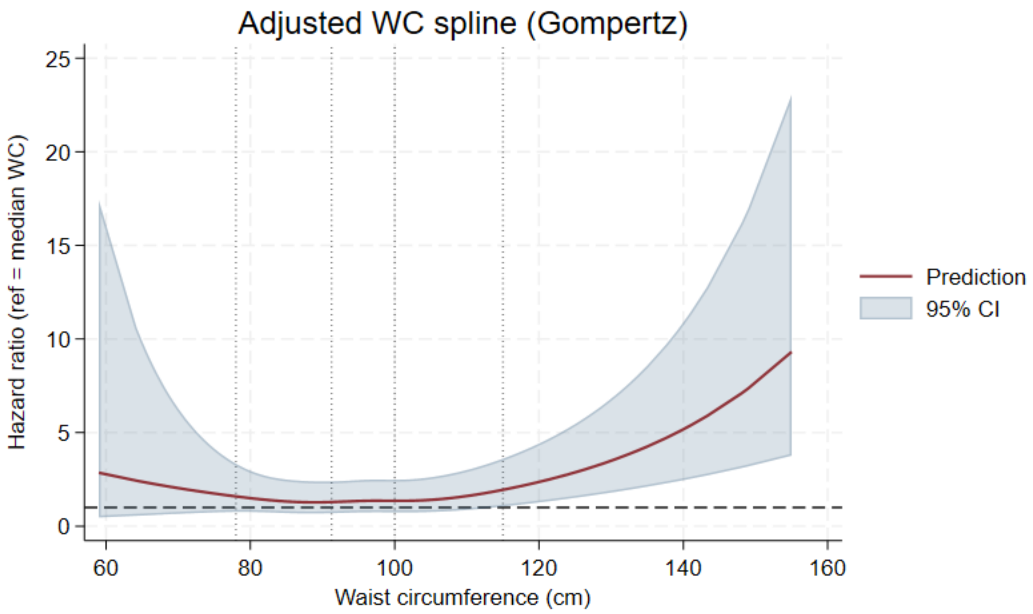

Legend: Adjusted hazard ratios (HRs) for incidence of CVD across the distribution of WC, estimated using restricted cubic splines from Gompertz proportional hazards models**.** HRs are shown relative to the median WC (100 cm). Dashed vertical lines indicate the locations of the four spline knots (85.5, 96.0, 103.0, and 116.75 cm). Shaded areas indicating 95% confidence intervals. Estimates at the extremes of the exposure distributions are based on fewer observations and should be interpreted with caution. Models were adjusted for covariates as described in the Methods.

Abbreviations: HR, hazard ratio; WC, waist circumference.

Supplementary Figure 6. Adjusted hazard ratios for the incidence of cardiovascular disease across body mass index

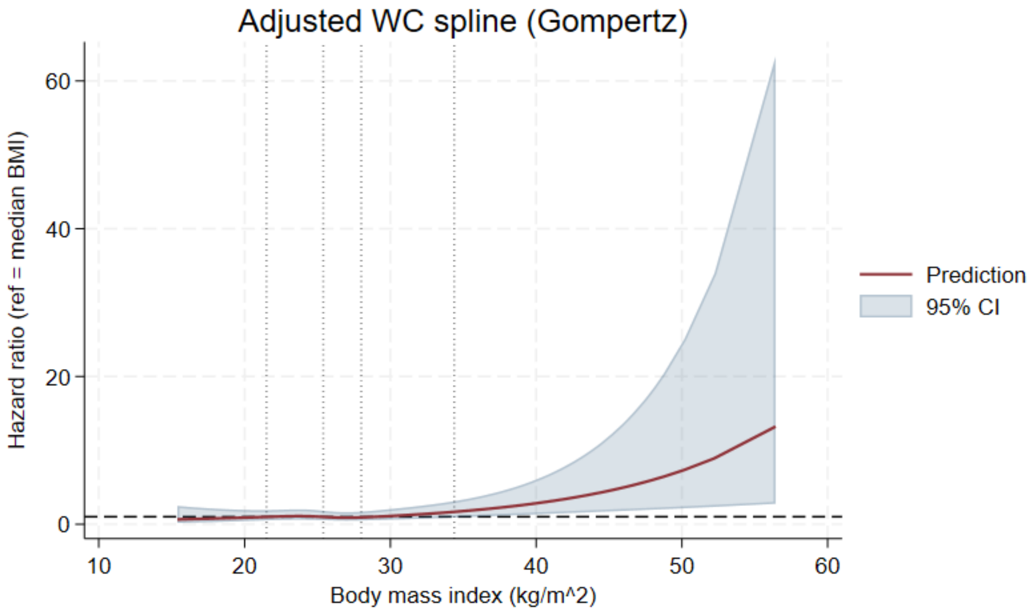

Adjusted hazard ratios (HRs) for incidence of CVD across the distribution of Wc, estimated using restricted cubic splines from Gompertz proportional hazards models**.** HRs are shown relative to the median BMI (26.74 kg/m^2). Dashed vertical lines indicate the locations of the four spline knots (22.40, 25.69, 27.87, and 32.74 kg/m^2). Shaded areas represent 95% confidence intervals. Estimates at the extremes of the exposure distributions are based on fewer observations and should be interpreted with caution. Models were adjusted for covariates as described in the Methods.

Abbreviations: HR, hazard ratio; BMI, body mass index.
